# Medical costs of a low skeletal muscle mass are modulated by dietary diversity and physical activity in community-dwelling older Taiwanese: a longitudinal study

**DOI:** 10.1186/s12966-017-0487-x

**Published:** 2017-03-14

**Authors:** Yuan-Ting C. Lo, Mark L. Wahlqvist, Yi-Chen Huang, Shao-Yuan Chuang, Chi-Fen Wang, Meei-Shyuan Lee

**Affiliations:** 1 School of Public Health, National Defense Medical Center, 161 Minchuan East Road, Sec. 6, Taipei, 11490 Taiwan, Republic of China; 2 Institute of Population Health Sciences, National Health Research Institutes, 35 Keyan Road, Zhunan, Miaoli County 35053 Taiwan, Republic of China; 3 Monash Asia Institute, Monash University, Caulfield East, PO Box 197, Melbourne, VIC 3145 Australia; 4 Graduate Institute of Life Sciences, National Defense Medical Center, 161 Minchuan Road, Sec. 6, Taipei, 11490 Taiwan, Republic of China

**Keywords:** Medical utilization, Older adults, Sarcopenia

## Abstract

**Background:**

Age-related loss of skeletal muscle mass (SMM) and function (sarcopenia) are associated with poor health outcomes and an economic burden on health care services. An appropriate diet and physical activity have been proposed for prevention and treatment of sarcopenia. Nevertheless, the effects on medical service utilization and costs remain unclear. This study determined the effects of SMM in conjunction with diet quality and physical activity on medical service utilization and expenditure in community-dwelling older Taiwanese.

**Methods:**

In total, 1337 participants from the Elderly Nutrition and Health Survey in Taiwan (1999–2000) were enrolled. An SMM index [SMMI, calculated by dividing SMM (kg) by height (m^2^)] was used as the marker of sarcopenia. Participants with the lowest SMMI quartiles (<11.4 kg/m^2^ for men and 8.50 kg/m^2^ for women) comprised the high-risk group, and the remainder comprised the low-risk group. Dietary information (dietary diversity: low and high) and physical activity (low and moderate) were obtained at baseline. Annual medical service utilization and expenditure were calculated from National Health Insurance claims until December 31, 2006. Generalized linear models were used to determine the association between the SMMI and annual medical service utilization and costs in conjunction with dietary diversity or physical activity.

**Results:**

After 8 follow-up years, regardless of gender, participants in the high-risk group reported significantly more hospitalization (days and expenditure) and total medical expenditure. Participants in the high-risk group who had low dietary diversity made fewer annual outpatient (14%), preventive care (19%), and dental (40%) visits, but exhibited longer hospitalization (102%) than did those who had a low SMMI and high dietary diversity. Similar patterns were observed in the corresponding medical expenditures. The findings were similar when considering physical activity. Being in the low-risk group in conjunction with having high dietary diversity or more physical activity was associated with the lowest annual adjusted mean hospitalization days with expenditure, and also total expenditure.

**Conclusions:**

A lower SMMI was associated with more hospitalization days and costs. However, high dietary diversity and more physical activity can attenuate the effects of lower SMMI on medical service utilization and expenditure.

## Background

Muscle mass loss is common in the elderly population, with an annual decline of 1%–2% after 50 years of age [1–4]. Several operational definitions for sarcopenia have been proposed; however, no consensus exists [5, 6]. Clinically, the view is now usually taken that a low skeletal muscle mass (SMM) has more utility if combined with a measure of muscle function and, together, this is referred to as sarcopenia [7]. While SMM does not include a measure of muscle function, muscle mass itself has relevance to nutritional status particularly in regard to nutrient reserves. It is, therefore, of interest to consider both SMM and sarcopenia as potential determinants of medical care usage.

Low SMM is associated with physical performance [8, 9], functional impairment [10], physical disability [10], chronic diseases [9], and mortality [11] in community-dwelling elderly people. The estimated direct health care cost attributable to sarcopenia in the United States in 2000 was about 1.5% (US$18.5 billion) of total health care expenditure for that year [12].

The first-line strategy for preventing and treating sarcopenia includes preserving SMM and maintaining muscle strength. Nutrition and exercise have been proposed for the prevention and management of age-related sarcopenia [13, 14]; however, the effectiveness of these interventions warrants additional study [15]. In single nutrient intake studies (e.g., protein, vitamins, minerals, and antioxidants) of sarcopenia [13, 15–23], the findings have been heterogeneous. In a few studies, foods and dietary patterns have been associated with sarcopenia [24–26]. Resistance exercises are effective for gaining lean body mass, thus inducing muscle hypertrophy and increasing muscle strength in older adults; but this may need to begin in early life [27, 28]. The effects of aerobic exercise or physical activity on muscle mass and strength in healthy elderly people are equivocal [29–31].

Those older Taiwanese with a low SMM have the highest mortality risk [11]. The effects of nonpharmacological strategies (diet or physical activity) in preventing SMM loss or treating sarcopenia are encouraging [25, 32, 33]. However, it is not known whether this reduces the health care burden and its expenditure. Therefore, the effects of SMM in conjunction with diet quality and physical activity on medical service utilization and expenditure in community-dwelling Taiwanese older adults were evaluated prospectively.

## Methods

### Population data sources

**Fig. 1:**
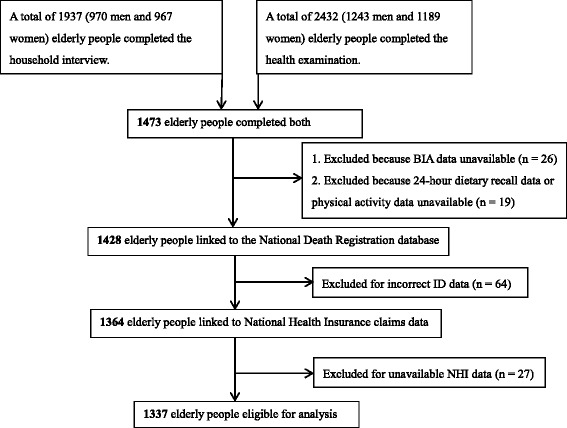
Flow chart

The Elderly Nutrition and Health Survey in Taiwan (NAHSIT), which was conducted between January 1, 1999 and December 31, 2000 was used. NAHSIT assessed the nutritional and health statuses of healthy Taiwanese people aged ≥ 65 years. It is a nationally representative survey incorporating a multistage, stratified, clustered probability sampling scheme. The detailed study design has been published elsewhere [34]. A total of 1473 participants completed a household interview and physical examination. We excluded 26 participants who did not complete the BIA and 19 whose diet or physical activity data were unavailable. Subsequently, 1428 participants were linked to the National Death Registration database until December 31, 2006 through personal identification numbers (IDs). Another 27 participants with incorrect IDs and 27 without National Health Insurance (NHI) records were excluded; thus, 1337 participants (689 men and 648 women) were finally enrolled. (Fig. 1). The original and analyzed data sets have been compared in a number of socio-demographic variables with no detectable difference found (data not shown). The ethics committees of both Academia Sinica and the National Health Research Institutes in Taiwan approved this study. All participants provided signed informed consent.

### National Health Insurance database

In 1995, Taiwan established the universal mandatory NHI program, which is financed through a means-related premium system and which covers more than 99% of the Taiwan population [35]. The NHI Research Database (NHIRD) provides claims data for reimbursement under the NHI program. The NHIRD encompasses complete records of medical service utilization and costs of the outpatient, inpatient, and emergency department claims of all beneficiaries [36].

### Skeletal muscle mass index

Dual-energy X-ray absorptiometry (DEXA) or bioelectrical impedance analysis (BIA) are commonly used to measure muscle mass and identify sarcopenia with low SMM [4, 37]. We used a BIA device (Parama-Tech BF-101) with two electric signals (right wrist and right ankle). All elders were fasted for more than 8 h, and assessed in the supine position. We used the resistance in OHMs from the device to estimate whole body SMM in kg by the formula, [0.401 × (height^2^/resistance) + (3.825 × gender) – (0.071 × age) + 5.102], which has been validated by magnetic resonance imaging in Taiwan among Chinese [38]. Absolute SMM was converted into an SMM index (SMMI) by dividing the height by meters squared (kg/m^2^) [11]. The quartiles (Q1–Q4) of SMMI for the total population were determined using the distributions for men and women. Participants with the lowest SMMI quartile (<11.4 kg/m^2^ for men and 8.50 kg/m^2^ for women) constituted the high-risk group; the low-risk group constituted participants with a relatively higher SMMI in accordance with the gender-specific distributions in Q2–Q4. This is supported by previous work in this population where the top three quartiles were clustered in so far as risk of mortality was concerned [11].

### Dietary information

Dietary quality was assessed using dietary diversity score (DDS) [39], which was calculated on the basis of a 24-h dietary recall obtained at baseline. The assessment comprised 6 food groups, namely dairy; eggs, beans, fish, and meat; rice and grains; fruits; vegetables; and fat and oil, in accordance with the Taiwanese Food Guides. Half a serving per day of one of the six food groups was required for a DDS score of 1, with total scores ranging between 0 and 6 [40]. DDSs of ≤ 4 and > 4 were considered as indicating low and high dietary diversity, respectively.

### Physical activity

A questionnaire was used to obtain the types and durations of sports and leisure activities per week for the participants. The metabolic equivalents (METs) per day were calculated for each activity by multiplying the corresponding METs and reported hours per day spent engaging in the activity. The total physical activity per participant was assessed by summing the daily METs of all activities. The cut off for acceptable levels, 1.5 MET, equivalent to 30 min of moderate physical activity per day, was based on US-CDC [41].

### Outcome measures

The claim data were obtained from the NHIRD for the period from the interview date of the participants until the date of death or December 31, 2006. Medical service utilization was defined as ambulatory care visits (four services: outpatient, preventive care, dental, and emergency services) and hospitalization days. The corresponding expenditure was considered the medical service expenditure. The ambulatory care visits were calculated using the frequency of visits, and the hospitalization days were the inpatient length of stay. In addition, medical expenditure was accounted for all resource inputs, including medical and surgical treatment inputs and contingent service fees. The total medical expenditure was the sum of ambulatory care and hospitalization expenditure. Furthermore, average annual utilization and expenditure were calculated by dividing the total expenditure by the follow-up years for each participant. Successive annual medical expenditures with an annual discount rate of 3% on the basis of an annual core consumer price index adjustment were used [42].

### Covariates

Covariates were obtained from the questionnaire at baseline. Demographic and socioeconomic status (SES) factors were gender, age (65–69, 70–74, 75–79, or ≥ 80 y), region of residence (*n* = 13: Hakka, mountainous areas, Eastern, Penghu, Northern 1–3, Central 1–3, Southern 1–3), ethnicity (Fukienese, Hakka, mainlander, or indigenous), education level (illiterate, primary or lower, or secondary education and higher), living status (alone or with others), self-reported financial status (enough, just enough, some difficulty, or very difficult), and household income (<15 000, 15 000-29 999, 30 000-49 999, or ≥ 50 000 NT$/month). Perceived health status was classified as good, fair, or poor. Activities of daily living (ADL) are the basic tasks of everyday life [43]. In total, nine questions regarding self-care task difficulty were asked, namely eating, moving between a bed and a chair, walking indoors and outdoors, dressing, bathing, toileting, and urinary and bowel continence. A score of 1 indicates any one of these difficulties. Multimorbidity was defined using the Charlson comorbidity index (CCI) [44] which we obtained at baseline from the 1999 NHI claims data from 1 year prior the interview for calculation.

### Statistical analysis

Categorical variables were reported by numbers and percentages, and continuous variables were expressed as means ± standard deviation. Categorical variables were compared using the chi-square test, and continuous variables were compared using one-way ANOVA. In addition, the mean and median are presented for medical service utilization and expenditure because of their right-skewed distributions. Multivariable generalized linear models, adjusted for potential covariates, with a log link and gamma distribution were used for assessing the association between high- and low-risk groups and medical service expenditure; a log link and Poisson distribution were used for determining medical service utilization [45]. The coefficients were exponentiated to obtain a ratio and percentage increase or decrease in ambulatory care visits, hospitalization days, and corresponding medical expenditure. The expenditure for participants who spent no money on medical expenditure was substituted using NT$0.01. Potential covariates were age, region of residence, ethnicity, education level, living status, self-reported financial status, household income (NT$/mo), perceived health status, ADL, CCI, energy (kcal/d), protein (g/d), DDS (≤4 or > 4), and physical activity (<1.5 MET/day or ≥1.5 MET/day). Each of these variables was either related to the exposure of interest (SMMI) or to outcomes (medical utilization or cost) or both. In terms of chronic disease, CCI was used as a collective index [46, 47]. SAS, Version 9.2 (SAS Institute Inc., Cary, North Carolina, USA) was used for all analyses. A two-tailed *P* < 0.05 was considered statistically significant.

## Results

All participants were followed for up to 8 years. The high-risk group had a lower SMM and SMMI than did the low-risk group (22.1 vs. 28.9 kg, *P* < 0.001; 8.88 vs. 11.7 kg/m^2^, *P* < 0.001). An age of ≥ 80 years, illiteracy, poor perceived health, higher ADL score, and a low CCI were observed for the participants in the high-risk group. The participants in the high-risk group had a lower percentage of high physical activity and DDS than did the participants in the low-risk group (Table 1).

**Table 1:** Baseline characteristics of participants with high and low risk stratified by SMMI^a^ (*n* = 1337)

		SMMI^a^		*P* value^c^
	% of sample	High risk	Low risk	
n (%)	1337	330 (24.7)	1007 (75.3)	
Skeletal muscle mass, kg	27.3 ± 7.40^b^	22.1 ± 6.02	28.9 ± 7.01	<0.001^d^
Skeletal muscle mass index, kg/m^2^	11.0 ± 2.23^b^	8.88 ± 1.68	11.7 ± 1.95	<0.001^d^
Gender, men, %	51.5	51.8	51.4	0.905
Age, y, %				
65-69	39.2	22.1	44.8	<0.001
70-74	33.7	33.0	33.9	
75-79	17.4	23.6	15.4	
≥ 80	9.72	21.2	5.96	
Ethnicity, %				
Fukienese	59.6	56.5	60.5	0.560
Hakka	10.6	10.6	10.6	
Mainlander	18.2	20.4	17.5	
Indigenous	11.6	12.5	11.3	
Personal education, %				
Illiterate	33.1	39.1	31.1	0.012
Primary and below	45.7	38.8	48.0	
Secondary	14.2	13.6	14.4	
Secondary and above	6.97	8.48	6.47	
Lived alone, %	9.42	11.5	8.74	0.134
Self-reported financial status, %				
Enough	14.1	12.3	14.7	0.530
Just enough	56.5	57.6	56.2	
Some difficulty	23.7	23.3	23.9	
Very difficult	5.70	6.92	5.30	
Household income, NT$/mo, %				
< 15,000	39.3	36.3	40.3	0.193
15,000–29,999	17.4	16.3	17.8	
30,000–49,999	19.8	25.3	18.0	
≥ 50,000	23.5	22.1	23.9	
Perceived health status, %				
Good	39.8	34.3	41.6	0.008
Fair	45.8	46.8	45.4	
Poor	14.4	19.0	12.9	
ADL	0.20 ± 1.07^b^	0.35 ± 1.37	0.15 ± 0.94	0.003^d^
CCI	3.64 ± 3.45^b^	3.19 ± 3.18	3.79 ± 3.52	0.006^d^
Body mass index	23.7 ± 3.65^b^	21.0 ± 3.22	24.6 ± 3.32	<0.001
Under weight	7.19	22.1	2.29	<0.001
Normal weight	45.7	61.8	40.5	
Over weight	29.1	11.5	34.9	
Obese	18.0	4.55	22.4	
DDS	4.44 ± 1.06^b^	4.36 ± 1.05	4.46 ± 1.07	0.142^d^
≤ 4	50.4	50.9	50.3	0.835
> 4	49.6	49.1	49.8	
Physical activity (MET/day)	2.11 ± 3.53^b^	1.93 ± 3.73	2.16 ± 3.46	0.284^d^
< 1.5	59.5	63.3	58.2	0.099
≥ 1.5	40.5	36.7	41.8	

Activities of daily living, *ADL* Charlson comorbidity index, *CCI* Dietary diversity score, *DDS* Skeletal muscle mass index, *SMMI* Metabolic equivalent, *MET*

^a^The high-risk group comprises participants with a lower SMMI (<11.4 kg/m^2^ for men and 8.50 kg/m^2^ for women)

^b^Mean ± SD

^c^Chi-square test

^d^One-way ANOVA

The participants in the low-risk group used more outpatient, preventive care, and dental services but fewer emergency services and had fewer hospitalization days (13.6 vs. 7.13 days, *P* < 0.001) compared with the participants in the high-risk group (Table 2). The participants in the high-risk group had lower annual outpatient expenditure (*P* < 0.01), preventive care (*P* < 0.05), dental services (*P* < 0 .01), but higher emergency (*P* < 0.01), hospitalization (NT$77 500 vs. 38 700, *P* < 0.001) and total medical expenditure (NT$102 000 vs. 67 400, *P* < 0.001). Both men and women in the high-risk group displayed similarly higher annual hospitalization (days and expenditure) and total medical expenditure compared to those with low-risk. Alternative analyses comparing Q1 to Q3 individually with Q4 do not alter these findings (data not shown).

**Table 2:** Multivariable generalized linear models for annual medical service utilization and expenditure stratified by high and low SMMI^a^ (*n* = 1337)

	Total (*n* = 1337)		Men (*n* = 689)		Women (*n* = 648)	
	High risk	Low risk	High risk	Low risk	High risk	Low risk
n (%)	330 (24.7)	1007 (75.3)	171 (24.8)	518 (75.2)	159 (24.5)	489 (75.5)
Deceased (%)	165 (50.0)	305 (30.3)	90 (52.6)	183 (35.3)	75 (47.2)	122 (25.0)
Medical service utilization						
Ambulatory care visits, times^b^						
Outpatient services	25.1 (21.9)^***^	28.7 (24.6)	25.4 (22.3)	26.8 (23.7)	24.7 (21.7)^***^	30.7 (25.3)
Preventive care	1.00 (0.93)	1.27 (1.16)	0.84 (0.82)	1.02 (0.98)	1.17 (1.08)	1.53 (1.46)
Dental services	0.65 (0.14)^***^	1.03 (0.42)	0.74 (0.14)^***^	1.11 (0.46)	0.56 (0.14)	0.94 (0.30)
Emergency	0.81 (0.31)^*^	0.57 (0.28)	0.83 (0.39)^*^	0.62 (0.29)	0.79 (0.29)	0.51 (0.27)
Hospitalization, days^b^	13.6 (2.15)^***^	7.13 (1.32)	16.0 (2.58)^***^	9.03 (1.45)	10.9 (1.52)^***^	5.12 (1.18)
Medical service expenditure, 1000 NT$^c^						
Ambulatory care^d^						
Outpatient services	20.8 (16.4)^**^	25.9 (18.4)	23.2 (17.1)	26.0 (18.1)	18.3 (15.9)^*^	25.7 (18.8)
Preventive care	0.21 (0.18)^*^	0.28 (0.24)	0.18 (0.15)	0.23 (0.19)	0.25 (0.20)^*^	0.34 (0.31)
Dental services	0.54 (0.07)^**^	0.88 (0.29)	0.63 (0.06)^*^	0.98 (0.36)	0.44 (0.07)	0.77 (0.24)
Emergency	2.80 (0.76)^**^	1.70 (0.51)	2.74 (0.80)^**^	1.77 (0.59)	2.87 (0.72)	1.62 (0.43)
Hospitalization^d^	77.5 (10.6)^***^	38.7 (6.89)	79.6 (12.2)^**^	49.8 (8.03)	75.3 (6.50)^***^	26.9 (5.86)
Total medical expenditure^d^	102 (34.4)^***^	67.4 (33.4)	106 (39.6)	78.8 (34.8)	97.2 (31.7)^**^	55.4 (32.2)

^a^The high-risk group comprises participants with a lower SMMI (<11.4 kg/m^2^ for men and 8.50 kg/m^2^ for women). The low-risk group comprises participants with a normal SMMI and serves as a reference group

^b^Mean (median). Outcome was assessed using GLMs with Poisson distribution

^c^NT$, with an exchange rate of approximately NT$31 to US$1 in 2017

^d^Mean (median). Outcomes was assessed using GLMs with gamma distribution

Models were adjusted for age, region of residence, ethnicity, education level, living status, self-reported financial status, household income (NT$/mo), perceived health status, ADL, CCI, energy (kcal/d), protein (g/d), DDS (≤4 or > 4), and physical activity (<1.5 MET/day or ≥ 1.5 MET/day)

^*^ *P* < 0.05, ^**^ *P* < 0.01, ^***^ *P* < 0.001

The joint effects of the SMMI (low vs. high) and DDS (low vs. high) on annual medical service utilization and expenditure after adjustment for covariates are listed in Table 3. The participants in the high-risk group who had a low DDS had high mortality rate and exhibited significantly fewer outpatient (14%), preventive care (19%), and dental (40%) service visits, but more emergency department visits (18%) and hospital stays (102%) compared with those in the low-risk group who had a high DDS. Similar patterns were observed for the corresponding medical expenditure.

**Table 3:** Multivariable generalized linear models for annual medical service utilization and expenditure stratified by SMMI^a^ and dietary diversity (*n* = 1337) (exp, β coefficients and 95% confidence intervals)

	SMMI high risk^a^		SMMI low risk		*P* for trend
	DDS ≤4	DDS >4	DDS ≤4	DDS >4	
	exp (β) (95% CI)	exp (β) (95% CI)	exp (β) (95% CI)		
n (%)	168 (12.6)	162 (12.1)	506 (37.9)	501 (37.5)	
Deceased n (%)	100 (59.5)	65 (40.1)	172 (33.9)	133 (26.5)	
Medical service utilization					
Ambulatory care visits, times^b^					
Outpatient services	0.86 (0.82, 0.89) ^***^	0.94 (0.91, 0.98) ^***^	0.94 (0.92, 0.96)^***^	Reference	<0.001
Preventive care	0.81 (0.68, 0.98) ^*^	0.92 (0.78, 1.10)	0.91 (0.81, 1.02)	Reference	0.030
Dental services	0.60 (0.47, 0.77) ^***^	0.76 (0.63, 0.92) ^**^	0.92 (0.81, 1.06)	Reference	<0.001
Emergency	1.18 (0.94, 1.48)	1.26 (1.01, 1.58)	1.02 (0.86, 1.22)	Reference	0.051
Hospitalization, days^b^	2.02 (1.90, 2.14) ^***^	1.70 (1.60, 1.80) ^***^	1.27 (1.21, 1,34) ^***^	Reference	<0.001
Medical service expenditure, 1000 NT$^c^					
Ambulatory care^d^					
Outpatient services	0.77 (0.66, 0.90) ^**^	0.85 (0.73, 0.99) ^*^	0.93 (0.84, 1.04)	Reference	<0.001
Preventive care	0.74 (0.65, 0.86) ^**^	0.92 (0.73, 1.05)	0.88 (0.80, 0.97)*	Reference	<0.001
Dental services	0.71 (0.54, 0.94) ^*^	0.77 (0.61, 0.97)^*^	0.88 (0.75, 1.05)	Reference	0.004
Emergency	1.42 (1.10, 1.83)^**^	1.53 (1.19, 1.96)^***^	1.37 (1.14, 1.64)^***^	Reference	<0.001
Hospitalization	2.24 (1.63, 3.07) ^***^	2.05 (1.48, 2.83) ^***^	1.27 (1.01, 1.58)^*^	Reference	<0.001
Total medical expenditure	1.51 (1.22, 1.87) ^***^	1.37 (1.11, 1.70) ^**^	1.12 (0.96, 1.29)	Reference	<0.001

^a^The high-risk group comprises participants with a lower SMMI (<11.4 kg/m^2^ for men and 8.50 kg/m^2^ for women). The low-risk group comprises participants with a normal SMMI

^b^Outcome was assessed using GLMs with Poisson distribution

^c^NT$, with an exchange rate of approximately NT$31 to US$1 in 2017

^d^Outcome was assessed using GLMs with gamma distribution

Models were adjusted for age, gender, region of residence, ethnicity, education level, living status, self-reported financial status, household income (NT$/mo), perceived health status, ADL, CCI, energy (kcal/d), protein (g/d), and physical activity (<1.5 MET/day or ≥ 1.5 MET/day)

^*^ *P* < 0.05, ^**^ *P* < 0.01, ^***^ *P* < 0.001

Table 4 shows the joint effect of SMMI (low vs. high) and physical activity (low vs. moderate-to-high) on annual medical service utilization and expenditure after adjustment for covariates. The participants in the high-risk group who reported low physical activity exhibited significantly fewer outpatient (9%) and dental (40%) service visits, but more emergency department visits (53%) and hospital stays (84%) compared with those in the low-risk group who engaged in more physical activity. Moreover, the participants in the high-risk group who reported low physical activity had lower outpatient, preventive care, and dental service expenditure, but longer hospitalization and total medical expenditure than did the participants in the low-risk group who engaged in more physical activity.

**Table 4:** Multivariable generalized linear models for annual medical service utilization and expenditure stratified by the SMMI^a^ and physical activity (*n* = 1337) (exp, β coefficients and 95% confidence intervals)

	SMMI high risk^a^		SMMI low risk		*P* for trend
	<1.5 MET/day	≥1.5 MET/day	<1.5 MET/day	≥1.5 MET/day	
	exp (β) (95% CI)	exp (β) (95% CI)	exp (β) (95% CI)		
n (%)	209 (15.6)	121 (9.05)	586 (43.8)	421 (31.5)	
Deceased n (%)	114 (54.6)	51 (42.2)	188 (32.1)	117 (27.8)	
Medical service utilization					
Ambulatory care visits, times^b^					
Outpatient services	0.91 (0.88, 0.94) ^***^	0.90 (0.86, 0.94) ^***^	0.96 (0.94, 0.99) ^**^	Reference	<0.001
Preventive care	0.86 (0.72, 1.02)	0.88 (0.72, 1.08)	0.93 (0.82, 1.04)	Reference	0.054
Dental services	0.60 (0.48, 0.75) ^***^	0.80 (0.65, 1.00)	0.93 (0.82, 1.06)	Reference	<0.001
Emergency	1.53 (1.23, 1.90) ^***^	1.03 (0.77, 1.37)	1.19 (0.99, 1.43)	Reference	<0.001
Hospitalization, days^b^	1.84 (1.73, 1.95) ^***^	1.73 (1.61, 1.85) ^***^	1.15 (1.10, 1.21) ^***^	Reference	<0.001
Medical service expenditure, 1000 NT$^c^					
Ambulatory care^d^					
Outpatient services	0.84 (0.73, 0.98) ^*^	0.86 (0.72, 1.02)	1.02 (0.91, 1.14)	Reference	0.008
Preventive care	0.82 (0.71, 0.93) ^**^	0.88 (0.76, 1.03)	0.92 (0.84, 1.01)	Reference	0.002
Dental services	0.68 (0.53, 0.88) ^**^	0.92 (0.71, 1.19)	1.01 (0.86, 1.19)	Reference	0.012
Emergency	1.24 (0.96, 1.59)	1.46 (1.08, 1.97)^*^	1.04 (0.87, 1.27)	Reference	0.018
Hospitalization	2.34 (1.74, 3.19) ^***^	2.26 (1.64, 3.40) ^***^	1.41 (1.13, 1.76)^**^	Reference	<0.001
Total medical expenditure	1.51 (1.23, 1.86) ^***^	1.37 (1.08, 1.74) ^**^	1.12 (0.96, 1.26)	Reference	<0.001

^a^The high-risk group comprises participants with a lower SMMI (<11.4 kg/m^2^ for men and 8.50 kg/m^2^ for women). The low-risk group comprises participants with a normal SMMI

^b^Outcome was assessed using GLMs with Poisson distribution

^c^NT$, with an exchange rate of approximately NT$31 to US$1 in 2017

^d^Outcome was assessed using GLMs with gamma distribution

Models were adjusted for age, gender, region of residence, ethnicity, education level, living status, self-reported financial status, household income (NT$/mo), perceived health status, ADL, CCI, energy (kcal/d), protein (g/d), and DDS (≤4 or >4)

^*^ *P* < 0.05, ^**^ *P* < 0.01, ^***^ *P* < 0.001

In addition, analysis was done by excluding 45 participants who died in the first year of follow-up and the results were similar (data not shown for the equivalents of Tables 3 and 4).

**Fig. 2:**
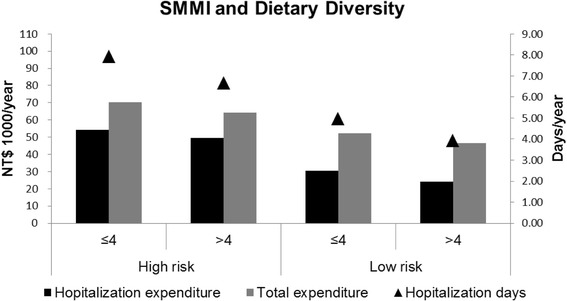
Annual adjusted mean hospitalization (days and expenditure) and total medical expenditure stratified by the SMMI and DDS (*n* = 1337). The models were adjusted for age (y), gender, region of residence, ethnicity, education level, living status, self-reported financial status, household income (NT$/mo), perceived health status, ADL, CCI, energy (kcal/d), protein (g/d), and physical activity (<1.5 MET/day or ≥ 1.5 MET/day)

The participants in the low-risk group who had a high DDS exhibited the fewest annual adjusted mean hospitalization days (3.92 days) and the lowest hospitalization expenditure (NT$24 200) as well as the lowest total medical expenditure (NT$46 600). The mean differences in annual hospital stays, expenditure, and total medical expenditure between the participants in the high-risk group who had a low DDS and in the low-risk group who had a high DDS were 3.99 days, NT$29 900, and NT$23 700, respectively (Fig. 2).

**Fig. 3:**
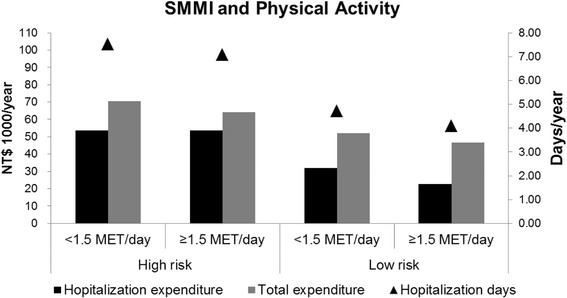
Annual adjusted mean hospitalization (days and expenditure) and total medical expenditure stratified by the SMMI and physical activity (*n* = 1337). The models were adjusted for age (y), gender, region of residence, ethnicity, education level, living status, self-reported financial status, household income (NT$/mo), perceived health status, ADL, CCI, energy (kcal/d), protein (g/d), and DDS (≤4 or > 4)

Participants in the low-risk group who reported moderate-to-high physical activity exhibited the shortest annual adjusted mean hospital stays (4.10 days) and the lowest hospitalization expenditure (NT$22 700) as well as the lowest total medical expenditure (NT$46 600). The mean differences in annual hospital stays, expenditure, and total medical expenditure between the participants in the high-risk group who had low physical activity and in the low-risk group who had more physical activity were 3.43 days, NT$30 800, and NT$23 900, respectively (Fig. 3).

Among the participants in the high-risk group, the annual adjusted mean hospitalization (days and expenditure) and total medical expenditure did not differ substantially between the low and the moderate-to-high physical activity sub-groups. In the same high-risk group, the differences in annual hospitalization days, expenditure, and total medical expenditure between the low and high DDS sub-groups were 1.25 days, NT$4 600, and NT$6 200, respectively (Figs. 2 and 3).

## Discussion

Regardless of gender, the older adults with a higher SMMI displayed a higher annual outpatient and dental service use, but fewer emergency department visits and less hospitalization (days and expenditure) and total medical expenditure than did those with a lower SMMI. Among these free-living older adults with a higher SMMI, those who also had a higher dietary diversity, had a lower annual emergency department care (visits and expenditure), inpatient (days and expenditure), and total medical expenditure. In addition, older adults with a higher SMMI and more physical activity were similarly advantaged. Nevertheless, dietary diversity had greater effects in saving health care usage and expenditure than did physical activity.

### Low skeletal muscle mass and health

Low SMM is associated with poor or impaired physical performance in community-dwelling elderly adults [8–10, 48, 49]. Physical performance is impaired because of weaker grip strength, slower gait speed, and poor mobility, which increase the risk of falling [50, 51]. Moreover, a low SMM is correlated with the metabolic syndrome [52], chronic kidney disease (CKD) [53], osteoporosis [54], and liver fibrosis [55]. Several endocrine diseases or CKD can accelerate the loss of muscle mass and strength, and lead to physical disability [56]. Elderly people with a low SMM or sarcopenia and chronic disease may experience a vicious cycle of functional decline, physical disability and loss of independence, on account of associated comorbidities [6], adding to health care resource utilization and expenditure. Disorders and diseases associated with low SMMI may result in limited mobility and impaired immune function, the latter on account of reduced substrate reserve for immunocompetence [57].

### Health care utilization and costs of sarcopenia

Health care utilization and costs associated with low SMM or sarcopenia are poorly defined. The estimated direct health care costs of sarcopenia in the United States in 2000 indicate that a 10% reduction in its prevalence would result in substantial annual savings [12]. Older participants with the lowest quartile of muscle density have a 51% higher risk of hospitalization than those in the highest quartile [58]. The length of hospital stay (LOS) is significantly longer in older patients with sarcopenia than in patients without sarcopenia (mean LOS, 13.4 versus 9.4 days, *P* = 0.003) [59]. In our study, participants with a lower SMMI had longer LOS along with greater hospitalization and total medical expenditures. Both men and women showed similar patterns. Hospitalization, even for a short time, is associated with an increased risk of subsequent functional decline, dependency, and disability in older patients [60, 61]. The annual difference in hospitalization costs between participants in the high- and low- risk groups was NT$38 800 (about US$1200) per person in the present study. This study has focused on direct health care expenditure; however, the public health and societal burden attributable to the loss of individual independence is substantial and also warrants attention [6].

### Preventive nutrition strategies to mitigate the public health burden

Limited evidence suggests that there is an association between foods or dietary patterns and outcomes related to muscle mass or function in community-dwelling older adults. In the Hertfordshire Cohort Study, higher fruit and vegetable, wholemeal cereal, and oily fish consumption were shown to be associated with better grip strength in community-dwelling older adults [24]. In a South Korean survey, vegetable consumption was directly associated with muscle mass in older women [62]. Iranian elderly in the highest tertile of the Mediterranean dietary pattern have lower odds for sarcopenia than those in the lowest tertile [26]. Thus, these foods or dietary patterns have been considered favorable in regard to muscle mass and strength, which our findings support. That a variety of foods in different settings is associated with muscle health is evident.

Few studies have reported the economics of sarcopenia or low SMM. We have investigated the medical costs of SMM in relation to dietary diversity. Participants with a lower SMMI and lower dietary diversity used more medical services, and had higher emergency, hospitalization, and total medical expenditures than did those with both higher.

The benefits of high dietary diversity on elderly health could be related to several factors. A highly diverse diet is rich in 6 food groups: dairy; eggs, beans, fish, and meat; rice and grains; fruits; vegetables; and fat and oils. By contrast, a poor diet is less diverse and, in our study population, was particularly insufficient in dairy, fruits, and vegetables [40]. Diets that can prevent sarcopenia have been found to be of better quality with relatively more protein, vitamin D, and antioxidants [23]. A food-based or whole diet approach, to reflect both quality and quantity rather than single nutrient supplements is more likely to be an effective prevention strategy [23, 63]. The benefits of sarcopenia avoidance together with a more diverse diet can also be seen in terms of population attributable risk (PAR) by way of lower hospitalization days (11.4%) and costs (13.5%), and total medical costs (6.04%) among older Taiwanese (data not shown).

There has been much interest in dairy products and protein, both whey and casein, as potentially protective against muscle loss, perhaps by way of the gut microbiome, peptide or leucine provision [64]. In those with high risk SMMI, DDS is of greater protective value than when this risk is lower. This might be explained by dairy intake. However, whether dairy is included in the DDS or not or whether dairy scores ranked at all or not, the impact of DDS in the high risk SMMI group was unchanged. Moreover, with inclusion of dairy in the model, the directions of the estimates for DDS groups reversed, which implies that dairy intake may behave as a negative confounder (data not shown). Thus, our study has demonstrated that non-dairy features of the DDS are principally responsible for its protective role in sarcopenia.

The association of physical inactivity with higher health care costs has not been convincingly related to sarcopenia [65–67]. In our study, low SMMI participants had greater hospitalization and total medical costs, regardless of their physical activity status. By contrast, a Japanese study demonstrated that high physical activity levels in elderly people resulted in the lowest total medical costs [68]. In general, for community-dwelling elderly people, exercise intervention seems to increase muscle mass and strength, and improve physical performance [15] with presumptive benefits to the health care system. Nevertheless, muscle mass and strength evident in later life may reflect not only the rate of loss with age, but also the peak attained earlier in life [69] with implications for prevention strategies through diet and physical activity [23].

### Limitations

First, we used BIA to measure SMM. This measurement is not as precise or accurate as that obtained through magnetic resonance tomography, computed tomography, or DEXA. Nevertheless, BIA is inexpensive and easy to perform in most settings, and the formula we used has been validated in several Chinese populations [38, 70]. Second, the DDS was derived from a 24-h dietary recall, which may not represent long-term dietary habits; however, community-dwelling older adults have relatively stable diets [40, 71]. Dietary patterns, like those based on several food groups, in our case 6 groups, may be stable from day-to-day while having different foods which contribute to the components of the pattern in question. This approach characterizes clinical nutrition practice (e.g., food group exchange). Such information is obtainable and recognizable from 24-h recalls. Even though our dietary methodology is imperfect, it has the ability to identify an association between diet and indices of health care system usage. Third, physical activity evaluation was restricted to sport and leisure time activities, but not occupation and will have been an underestimation [67]. Nevertheless, we have assessed the correlations between our index of physical activity and related phenomena, such as physical functioning from SF-36, ADL and perceived physical activity, which indirectly support the utility of our physical activity measurement (data not shown). It remains possible that we have underestimated the role of physical activity in health care system usage for methodological reasons. Finally, out-of-pocket (OOP) payments are required for some medical services, pharmaceuticals, and devices not covered by the NHI program. Household income is positively associated with health care utilization and OOP payments [46], which can underestimate medical costs, particularly in high-income households; for this reason, we adjusted for SES.

## Conclusions

A higher SMMI in later life is associated with shorter hospitalization and total medical expenditure. A more diverse diet can offset the consequent increased financial burden in the health care system. Although less evident, there is similar potential with physical activity in regard to lower SMMI. The use of non-pharmacological strategies such as diet and physical activity to prevent or decelerate the progression of sarcopenia may, however, need a life-long approach for optimal effect.

## Abbreviations

ADL: Activities of daily living
BIA: Bioelectrical impedance analysis
CKD: Chronic kidney disease
DDS: Dietary diversity score
DEXA: Dual-energy X-ray absorptiometry
LOS: Length of hospital stay
METs: Metabolic equivalents
NAHSIT: Nutrition and health survey in Taiwan
NHIRD: NHI research database
SES: Socioeconomic status
SMM: Skeletal muscle mass
SMMI: Skeletal muscle mass index
